# Research–evidence gap analysis of hypofractionated radiotherapy for breast cancer: a bibliometric knowledge graph study

**DOI:** 10.3389/fonc.2026.1746949

**Published:** 2026-02-26

**Authors:** Bingyu Liu, Junbao Xu, Jianing Wang, Feng Liu, Hui Xing, Shuai Wang, Xiaohua Zhao, Longgang Wang

**Affiliations:** 1 School of Clinical Medicine, Shandong Second Medical University, Weifang, Shandong, China; 2 Faculty of Medical Images, Shandong Second Medical University, Weifang, Shandong, China; 3 Interventional Vascular Surgery Center, Affiliated Hospital of Shandong Medical University, Weifang Shandong, China; 4 Department of Radiotherapy, School of Medical Imaging, Affiliated Hospital of Shandong Second Medical University, Shandong Second Medical University, Weifang, Shandong, China; 5 Department of Thoracic Surgery, Affiliated Hospital of Shandong Second Medical University, Weifang, Shandong, China; 6 Shandong Cancer Hospital and Institute, Shandong First Medical University and Shandong Academy of Medical Sciences, Jinan, China

**Keywords:** bibliometrics, breast cancer, evidence gap, hypofractionated radiotherapy (HFRT), knowledge graph

## Abstract

**Background:**

Hypofractionated radiotherapy (HFRT) has become a standard option for early-stage breast cancer, supported by multiple randomized controlled trials and endorsed by major guidelines. However, whether research production, high-level evidence, and clinical guideline adoption are fully aligned remains unclear.

**Methods:**

Publications on HFRT for breast cancer (2014–2024) were retrieved from the Web of Science Core Collection. Bibliometric analyses were performed using CiteSpace (6.4.R1) and VOSviewer (1.6.20) to assess publication trends, collaboration networks, keyword clustering, and highly cited references. In parallel, clinical trials from PubMed were reviewed to evaluate the evolution of clinical evidence. A knowledge-mapping framework was applied to identify research evidence gaps.

**Results:**

A three-phase developmental pattern was identified: slow growth (2014–2019), rapid expansion (2019–2021), and stabilization (2021–2024). Italy, the United States, and China accounted for more than 60% of global publications. Research hotspots shifted from efficacy validation to toxicity, dose optimization, and patient-centered outcomes. Although efficacy evidence is well established, significant gaps persist, including (i) limited long-term cardiac and pulmonary toxicity data, (ii) inconsistent cosmetic and quality-of-life reporting—especially among younger patients, and (iii) insufficient evidence for locally advanced disease or post-reconstruction irradiation.

**Conclusions:**

HFRT research has matured substantially and strongly supports its use in early-stage breast cancer. However, long-term safety, patient-reported outcomes, and complex clinical scenarios remain underexplored. Future work should prioritize multicenter prospective studies, integration of radiomics and AI-driven analyses, and standardized reporting to better align research output with guideline development and patient needs.

**Systematic review registration:**

[website], identifier registration number.

## 1 Introduction

Breast cancer remains the most common malignancy among women worldwide and a leading cause of cancer-related mortality. Radiotherapy plays a central role in breast-conserving therapy, with fractionation schedules directly influencing tumor control and treatment-related toxicity. In recent years, hypofractionated radiotherapy (HFRT) has emerged as a standard alternative to conventional fractionation, supported by pivotal randomized controlled trials—such as the UK START trials, FAST-Forward, and the Canadian Whelan study—and subsequently incorporated into major international guidelines, including those from ASTRO, ESTRO, NCCN, and CSCO (1). Despite the growing body of evidence and increasing clinical adoption of HFRT, discrepancies remain between research activity, available high-level clinical evidence, and guideline recommendations. In particular, several clinically relevant areas continue to lack comprehensive and consistent evidence, including long-term cardiac and pulmonary toxicity, patient-reported cosmetic and quality-of-life outcomes, and the application of HFRT in complex clinical scenarios such as locally advanced disease or post-reconstruction radiotherapy (2–5). Previous bibliometric studies have broadly examined breast cancer or radiotherapy-related research; however, they have rarely focused on the alignment between research output, clinical evidence maturation, and guideline adoption in the context of HFRT. Therefore, the present study applies bibliometric and knowledge-mapping approaches to systematically characterize global research trends in HFRT for breast cancer and to identify critical research–evidence gaps. By integrating publication patterns with clinical trial evidence and guideline positions, this study aims to provide a structured overview of the field and to inform future research priorities and evidence translation (6, 7).

## 2 Data and methods

### 2.1 Data sources

A comprehensive literature search was conducted in the Web of Science Core Collection (Science Citation Index Expanded, SCI-E) for publications between January 1, 2014, and December 31, 2024. The search strategy was as follows:

TS=(“hypofractionated radiotherapy” OR “hypofractionation” OR “hypofractionated radiation therapy”)AND TS=(“breast cancer”).

Document types were limited to Article and Review. Only studies published in English were included. A total of 328 articles were obtained.

To ensure the completeness and reliability of the retrieved literature, all publications were retrieved from the Web of Science Core Collection (SCI-E) and the following filtering and deduplication strategies were applied:

Deduplication: Duplicate records were removed using the built-in deduplication feature of Web of Science to avoid repetition of publications, which could interfere with the analysis.

Exclusion Criteria: To ensure the rigor of the analysis, the following types of publications were excluded:

Conference abstracts: Only peer-reviewed full-text journal articles and reviews were included. Non-original research: Case reports, reviews, and non-clinical studies were excluded as they do not contribute new evidence directly relevant to our analysis.

Database Selection: Web of Science Core Collection was selected as the sole data source due to its comprehensive coverage of high-quality journals globally, especially in the medical and life sciences fields. While cross-database searches could increase the breadth of data, this study focused on deep analysis of the Web of Science database. Additionally, Web of Science is considered highly reliable in the fields of medicine and life sciences, encompassing a vast number of influential journals and publications. Time Range (2014–2024): The time range of 2014–2024 was chosen for the following reasons:

Guideline Shifts: After 2014, several major international clinical guidelines (e.g., ASTRO, ESTRO, NCCN) began recommending hypofractionated radiotherapy (HFRT) as a standard treatment for breast cancer, and research from this period directly influenced clinical practice.

Key Studies (e.g., FAST-Forward): Landmark trials such as the FAST-Forward study, published in 2019, provided strong evidence for the use of HFRT. The period from 2014 to 2024 reflects the rapid growth of research in this field, and thus was selected as the time window for this study.

### 2.2 Bibliometric tools and analysis

This study systematically searched the literature on hypofractionated radiotherapy for breast cancer from 2014 to 2024. Following the systematic retrieval strategy, data were analyzed using two specialized bibliometric tools: CiteSpace (6.4.R1) and VOSviewer (1.6.20) (8, 9). Using CiteSpace (6.4.R1) software with a 1-year time slice, node types encompassed keywords, institutions, countries, and authors (10–12). The Pathfinder + Pruning sliced networks algorithm was applied for network pruning. Emergent word analysis identified research frontiers, and knowledge maps depicting keyword co-occurrence and literature co-citation were generated (13, 14). Simultaneously, VOSviewer (1.6.20) was employed to visualize research hotspots, developmental trajectories, and collaborative relationships through keyword co-occurrence clustering, systematically outlining the overall development of large-field fractionated radiotherapy for breast cancer over the past decade (12). Figures were generated using CiteSpace (6.4.R1) and VOSviewer (1.6.20) (15, 16).

### 2.3 Analysis content

1. Annual Publication Trends: We calculated the annual volume of breast cancer large-field radiotherapy-related literature indexed in the Web of Science Core Collection from 2014 to 2024, plotting a line graph to illustrate growth trends. Descriptive statistical analysis identified growth phases and associated them with key events during the same period.
2. National/Institutional/Author Collaboration Networks: Using CiteSpace (6.4.R1) to set up “country,” “institution,” and “author” nodes, social network analysis calculates degree centrality and intermediary centrality to identify major contributing countries (e.g., the United States, China, and the United Kingdom as core producing nations), key research institutions (e.g., Cancer Hospital of the Chinese Academy of Medical Sciences, Memorial Sloan Kettering Cancer Center in the United States, and the University of Milan in Italy); Visualize collaboration networks to analyze cooperation intensity and patterns between countries (e.g., China-US, China-UK), institutions (e.g., universities vs. hospitals, research institutes vs. clinical centers), and authors, revealing global collaboration structures and core collaborative groups.
3. Keyword Clustering and Emergent Terms: Extract keywords from article titles, abstracts, and author affiliations; perform keyword co-occurrence analysis using VOSviewer (1.6.20); identify emergent keywords over the past decade (2014-2024) via CiteSpace (6.4.R1) emergent term detection to reveal emerging hotspots and research frontiers within the field.
4. Highly Cited Literature and Research Frontiers: Using CiteSpace (6.4.R1) and VOSviewer (1.6.20) to screen highly cited literature, combined with the content of highly cited papers and emergent keyword results, to distill current research frontiers, providing references for subsequent research priorities and clinical guideline updates.

## 3 Research findings

### 3.1 Publication trends

**Figure 1:**
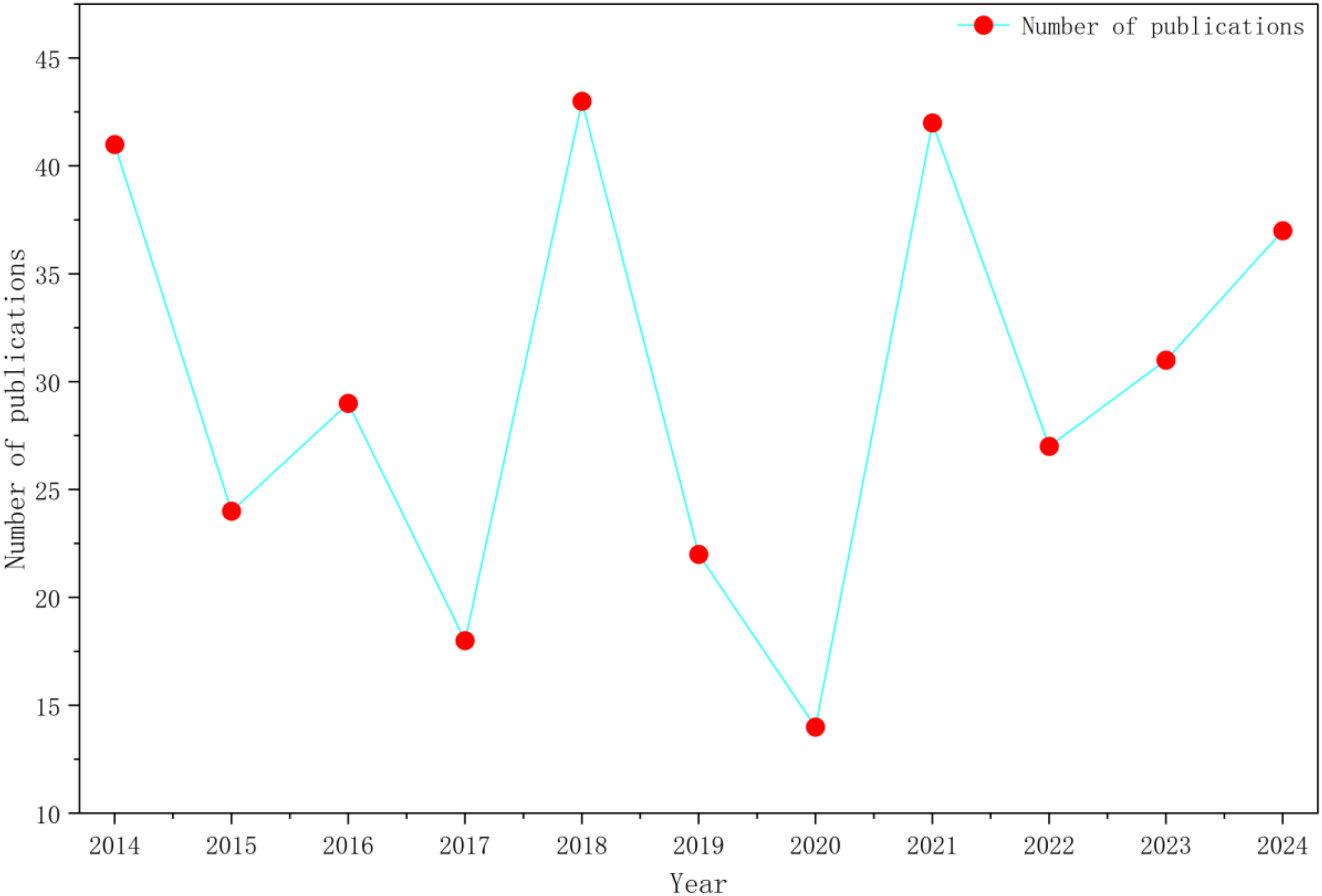
Annual number of publications on hypofractionated radiotherapy for breast cancer, 2014–2024.

Based on the Web of Science database, data screening and visualization yielded **Figure 1**.

**Figure 1** illustrates the annual number of publications on hypofractionated radiotherapy (HFRT) for breast cancer from 2014 to 2024 (17). Overall, the publication output demonstrates a fluctuating but upward trajectory, reflecting the progressive maturation of this research field. From 2014 to 2017, the number of publications remained relatively modest, ranging from 18 to 41 articles per year. This period represents an initial exploratory phase, during which hypofractionated radiotherapy had not yet been widely adopted in routine clinical practice, and evidence was mainly derived from early-phase studies and subgroup analyses of randomized trials (18–20). A marked increase was observed in 2018, when annual publications peaked at 43 articles. This surge likely reflects growing academic interest driven by accumulating clinical evidence and the dissemination of landmark trial results, which reinforced the non-inferiority of HFRT compared with conventional fractionation in early-stage breast cancer. Subsequently, publication output declined between 2019 and 2020, reaching a nadir of 14 articles in 2020. This temporary reduction may be attributable to a combination of factors, including the consolidation phase following major efficacy trials, the shift toward long-term follow-up studies requiring extended observation periods, and disruptions to clinical research activity during the COVID-19 pandemic (21). From 2021 onward, the number of publications increased again, with a peak of 42 articles in 2021 and a sustained rise through 2024. This rebound suggests a transition into a more mature research stage, characterized by a shift in focus from efficacy validation toward toxicity profiling, dose optimization, patient-reported outcomes, and the integration of advanced techniques such as image-guided radiotherapy and artificial intelligence–assisted planning. Taken together, the observed publication trend supports a three-phase developmental pattern of HFRT research in breast cancer: an initial exploratory phase (2014–2017), a rapid expansion phase (2018–2021), and a stabilization and refinement phase (2022–2024). This evolution mirrors the gradual translation of HFRT from clinical research into guideline-supported standard practice (22–26).

### 3.2 Publication frequency by authors

**Figure 2:**
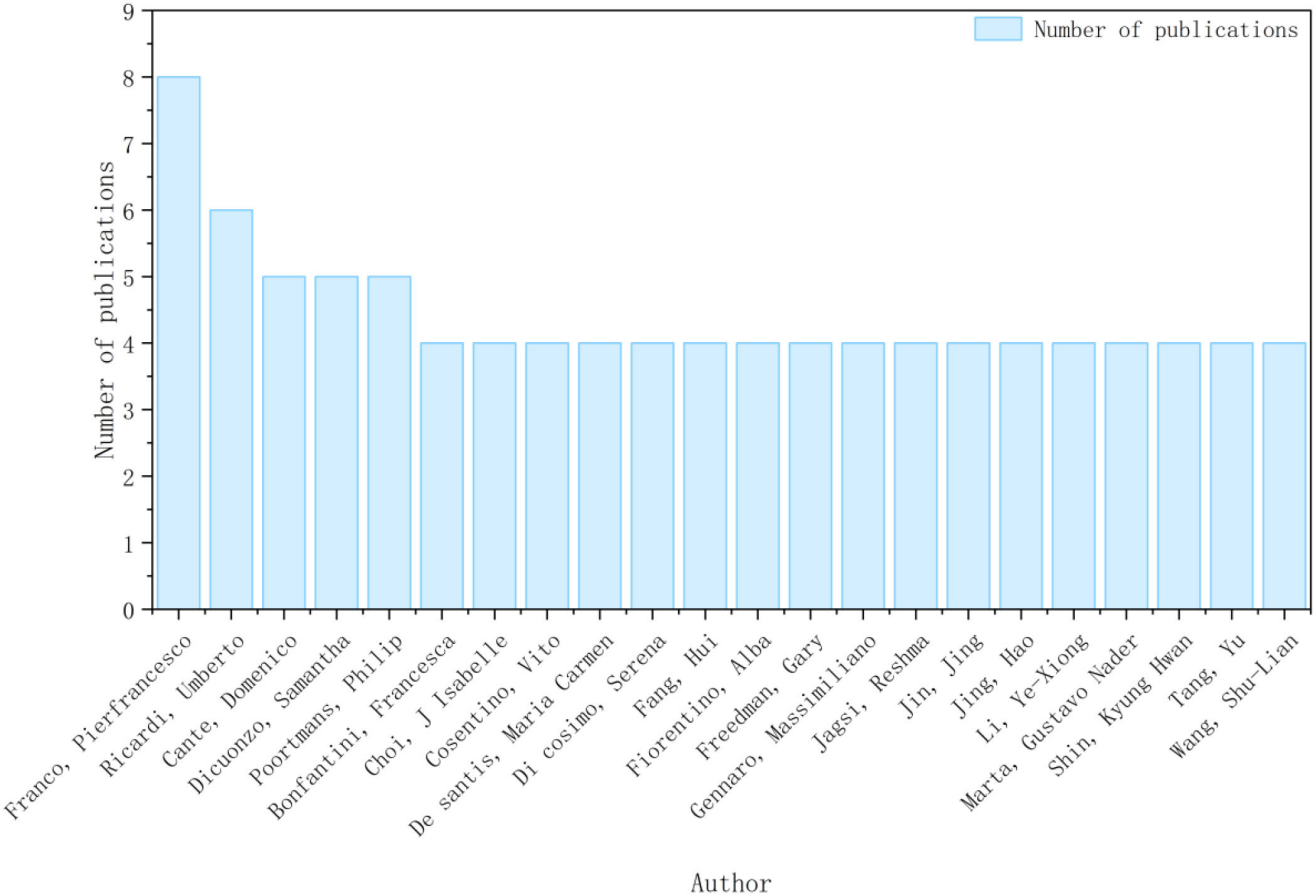
Publication count of authors (≥4 publications).

From 2014 to 2024, a total of 328 authors contributed to research on high-dose fractionated radiotherapy for breast cancer. Each author published between 1 and 8 papers. Due to the large number of authors, only those with at least 4 publications were included in the analysis and visualization, as shown in **Figure 2**.

**Figure 2** presents the distribution of publication output among the most productive authors (≥4 publications) in the field of hypofractionated radiotherapy for breast cancer between 2014 and 2024. Overall, author productivity shows a relatively balanced pattern, with no single author overwhelmingly dominating the research output. The most prolific author contributed eight publications, while a small number of authors published five to six articles. The majority of included authors produced three to four publications during the study period, indicating a moderately distributed authorship structure rather than a highly centralized one. This pattern suggests that research on hypofractionated radiotherapy for breast cancer is driven by multiple active research groups rather than a single dominant team. Such a decentralized authorship structure is consistent with the multicenter and collaborative nature of clinical radiotherapy research, particularly in fields reliant on large randomized trials and long-term follow-up. Furthermore, the relatively narrow range of publication counts among leading authors reflects the maturity of the field, where research contributions are widely shared across institutions and countries, rather than concentrated within a limited number of individual investigators.

**Figure 2** also illustrates the publication output of authors who contributed three or more articles to research on hypofractionated radiotherapy for breast cancer between 2014 and 2024. A total of 22 high-productivity authors were identified, reflecting the core group of researchers actively engaged in this field. Among these authors, Pierfrancesco Franco ranked first with eight publications (27),followed by Riccardo Umberto with six publications. Several authors, including Cante Domenico, Dicuonzo Samantha, Poortmans Philip, and Bonfantini Francesca, each contributed five publications, indicating sustained research involvement over the study period. The majority of the remaining authors—such as Choi Francesca, Cosentino Isabelle, De Santis Maria Carmen, Di Cosimo Serena, Fang Hui, Fiorentino Alba, Freedman Gary, Gennaro Massimiliano, Jagsi Reshma, Jing Jing, Jing Hao, Li Yexiong, Marta Gusevskaya, Shin Kyung Hwan, Tang Yu, and Wang Shu-Lian—published four articles each. This relatively narrow range of publication output suggests a balanced authorship structure without excessive concentration of contributions among a small number of individuals. Overall, the author productivity distribution indicates that research on hypofractionated radiotherapy for breast cancer is driven by multiple active investigators and collaborative research groups rather than by a single dominant author. Such a pattern is consistent with the multicenter, multidisciplinary nature of clinical radiotherapy research and reflects the maturity of this research field (28–30).

Only authors with four or more publications were included to improve network clarity and interpretability.

**Figure 3:**
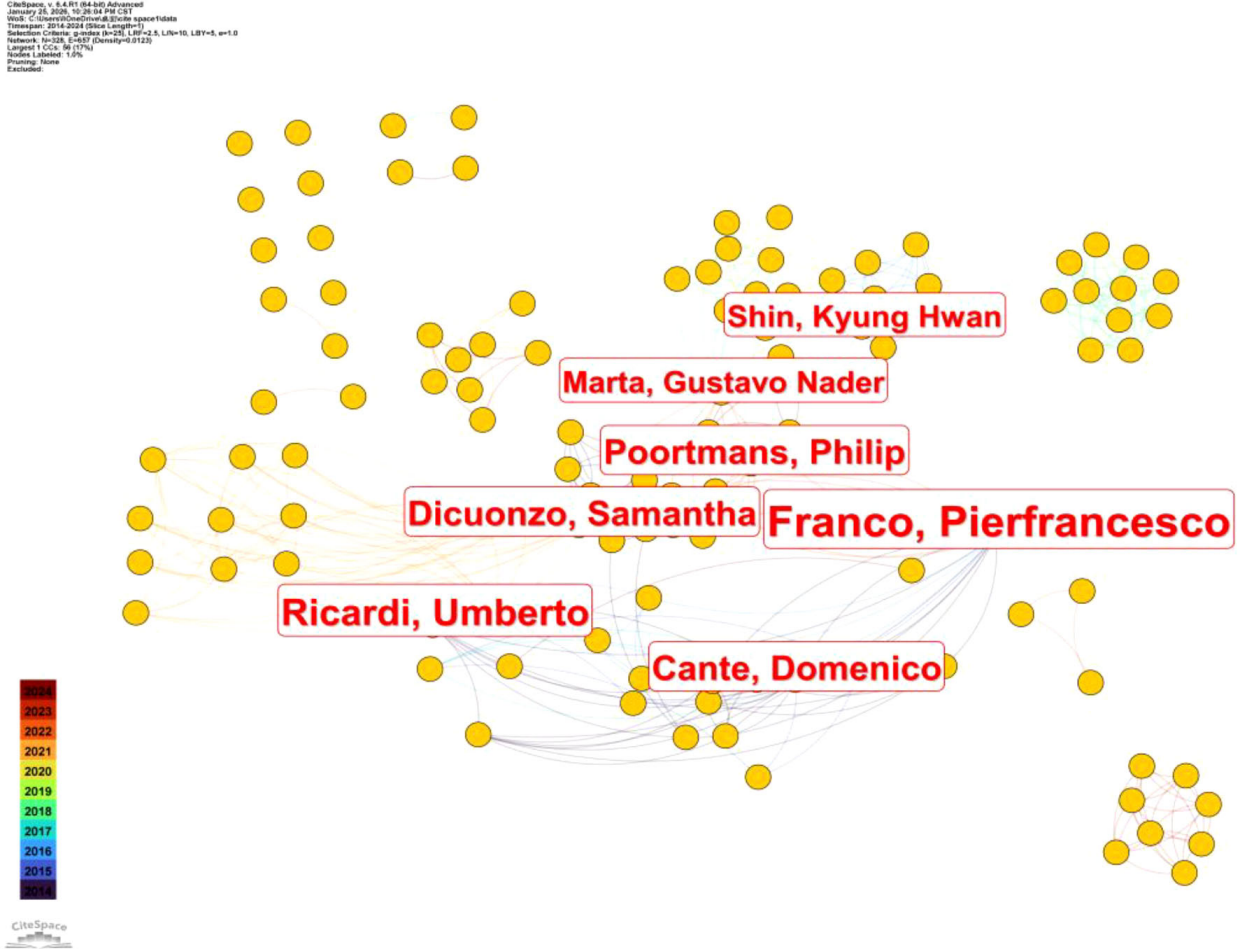
Author collaboration network of researchers.

**Figure 3** illustrates the author collaboration network in research on hypofractionated radiotherapy for breast cancer from 2014 to 2024. Each node represents an author, while links indicate co-authorship relationships, with larger nodes reflecting higher publication output. The network reveals a moderately connected structure characterized by several prominent collaborative clusters. Among the identified authors, Pierfrancesco Franco occupies a central position within the collaboration network, indicating extensive cooperative relationships with multiple research groups. Authors such as Philip Poortmans, Domenico Cante, Samantha Dicuonzo, and Umberto Ricardi are closely connected to Franco, forming a core European collaboration cluster primarily focused on clinical applications and outcome evaluation of hypofractionated radiotherapy. Additional collaborative subclusters are observed around authors including Marta Gustavo Nader and Kyung Hwan Shin, suggesting regional or institutional research teams that maintain active internal collaboration while also engaging in broader international networks. These clusters highlight the role of multicenter cooperation in advancing hypofractionated radiotherapy research, particularly in studies requiring large patient cohorts and long-term follow-up. Overall, the author collaboration network demonstrates that research in this field is not dominated by isolated investigators but is instead driven by interconnected research groups. This collaborative structure is consistent with the multidisciplinary and multicenter nature of radiotherapy research and supports the robustness and maturity of the research landscape in hypofractionated radiotherapy for breast cancer (31).

### 3.3 Countries and institutions

**Figure 4:**
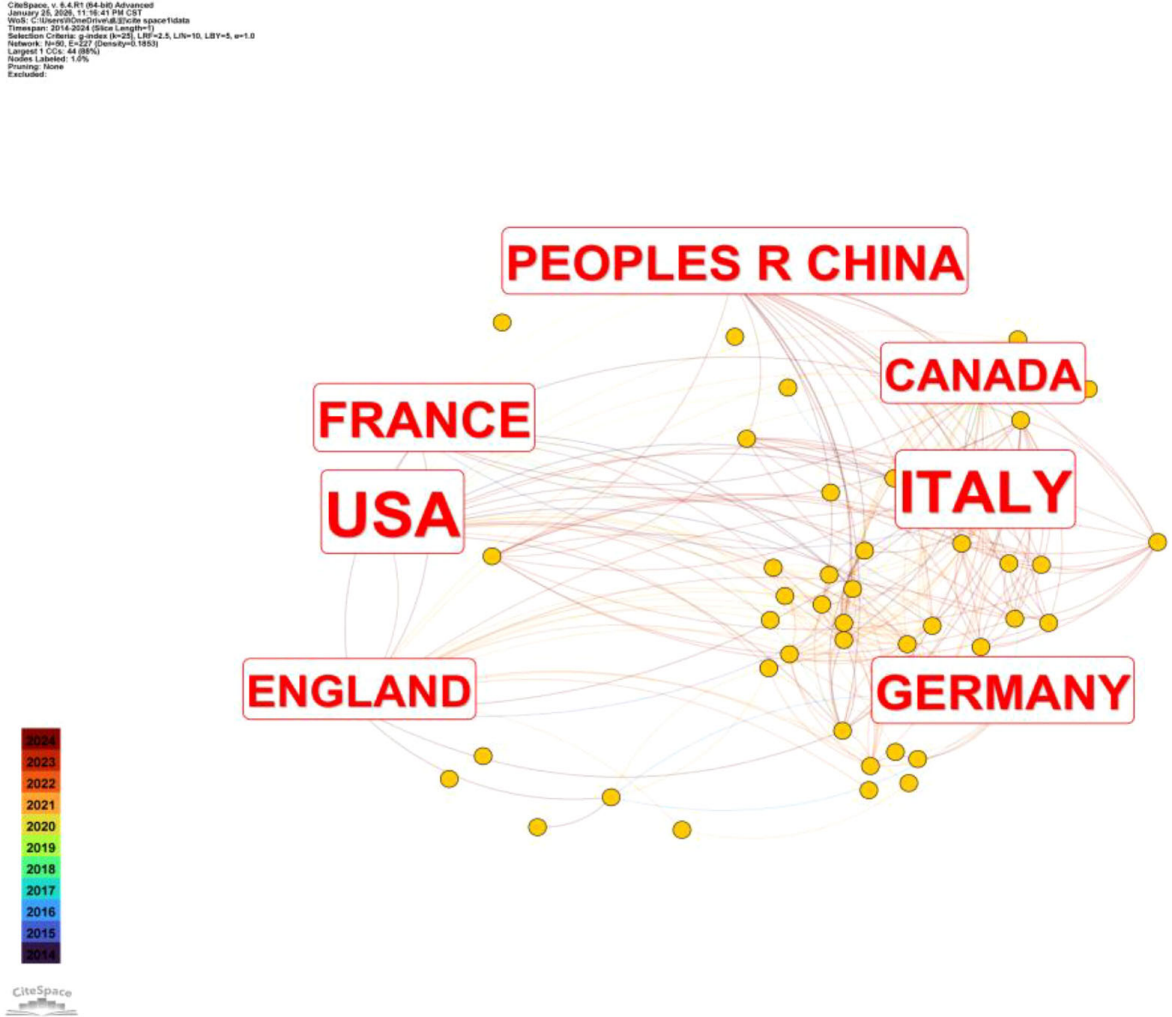
Visual analysis of national publication output.

**Figure 5:**
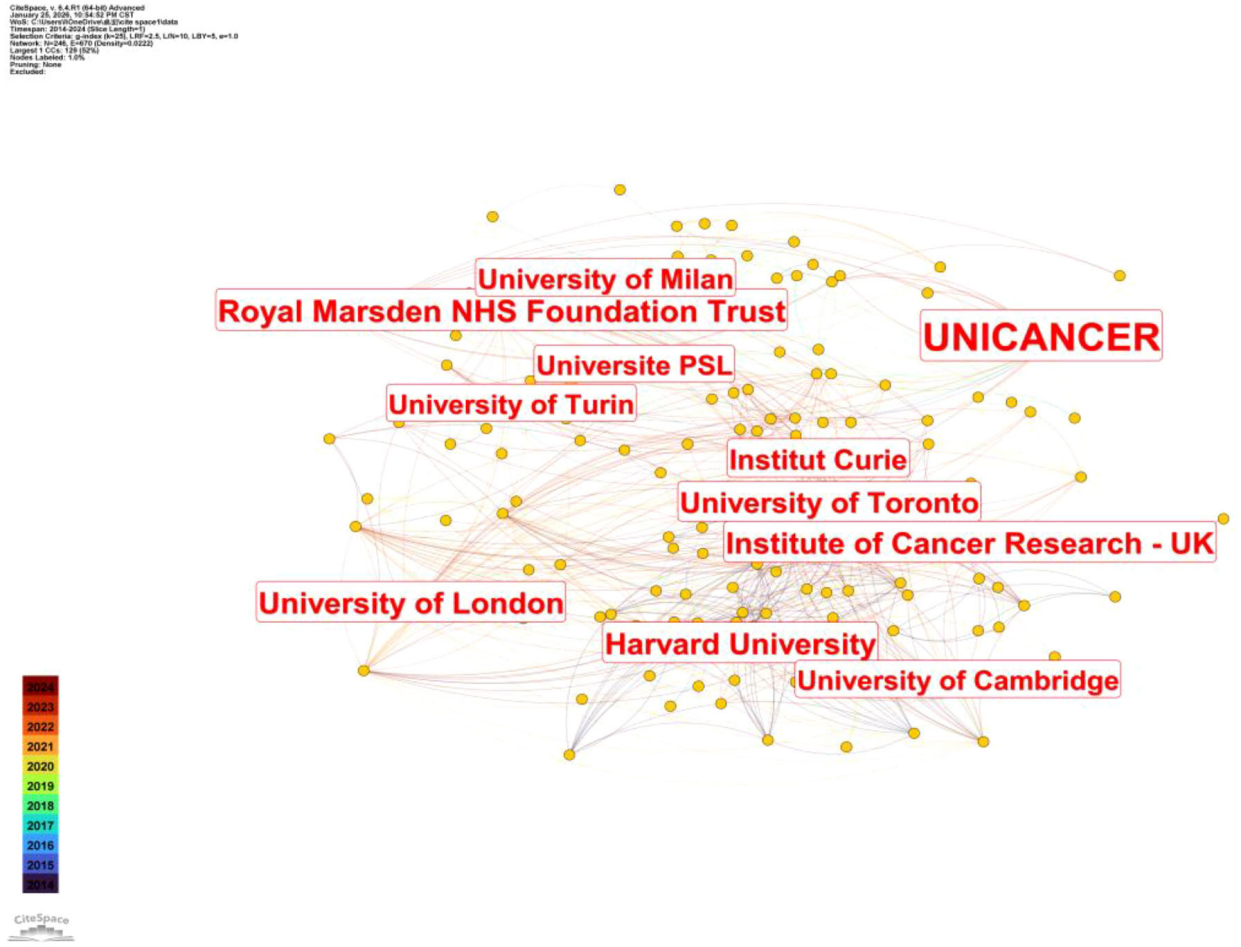
Visualization of global institutional publication output.

Statistics show that 61 countries worldwide participated in research on hypofractionated radiotherapy for breast cancer. Due to the large number of countries, only the top ten countries by publication volume are listed. The success and global adoption of hypofractionated radiotherapy fundamentally exemplify international scientific collaboration. No single nation could have achieved this alone. It was through cross-border collaboration that the efficacy of this therapy was rapidly and robustly validated, transforming global clinical practice. See **Figures 4**, **5**.

**Figure 4** presents the international collaboration network of countries involved in research on hypofractionated radiotherapy for breast cancer from 2014 to 2024. Each node represents a country, and the links between nodes indicate international co-authorship relationships. Larger nodes reflect higher publication output, while denser connections indicate stronger collaborative intensity. The network reveals a highly interconnected global research structure, with Italy, the United States, Germany, England, France, Canada, and the People’s Republic of China forming the core contributing countries. Among them, Italy and the United States occupy central positions in the network, demonstrating extensive collaborative links with multiple countries and acting as major hubs of international cooperation. Germany, England, and France also show strong connectivity, particularly within European collaborative networks, reflecting the long-standing tradition of multicenter clinical trials and cross-border research initiatives in radiotherapy. Canada is closely linked with both European countries and the United States, consistent with its active participation in landmark randomized trials in breast cancer radiotherapy. Notably, the People’s Republic of China has emerged as an increasingly connected node in the network, engaging in collaborations with several high-output countries, including the United States and European nations. This pattern indicates the growing international integration of Chinese research groups in hypofractionated radiotherapy studies over the past decade. Overall, the country collaboration network highlights the global and cooperative nature of research in hypofractionated radiotherapy for breast cancer. The strong interconnections among leading countries support the development of high-quality multicenter evidence and facilitate the translation of research findings into international clinical guidelines (32–34).

**Figure 5** illustrates the institutional collaboration network in research on hypofractionated radiotherapy for breast cancer from 2014 to 2024. Each node represents an institution, and links between nodes indicate inter-institutional co-authorship. Larger nodes reflect higher publication output, while denser connections indicate stronger collaborative relationships. The network demonstrates a highly interconnected institutional structure, with several prominent academic and clinical centers serving as major hubs. Among them, the University of Milan, UNICANCER, Institut Curie, University of Toronto, University of Cambridge, Harvard University, and the Institute of Cancer Research (UK) occupy central positions, indicating their substantial contributions and extensive collaborative activities. European institutions form a particularly dense collaboration cluster, including the University of Milan, University of Turin, Université PSL, Institut Curie, Royal Marsden NHS Foundation Trust, and University of London. This pattern reflects the strong tradition of multicenter clinical trials and coordinated research frameworks in European radiotherapy research. In parallel, North American institutions such as Harvard University and the University of Toronto are closely integrated into the global collaboration network, frequently partnering with leading European centers. These transcontinental collaborations play a critical role in generating high-quality clinical evidence and facilitating the international dissemination of research findings. Overall, the institutional collaboration network highlights the pivotal role of major academic hospitals and comprehensive cancer centers in advancing hypofractionated radiotherapy research for breast cancer. The extensive inter-institutional connections underscore the multicenter and cooperative nature of this field, which is essential for conducting large-scale clinical trials and achieving long-term follow-up outcomes (35–37).

### 3.4 Keyword clustering and emergence

**Figure 6:**
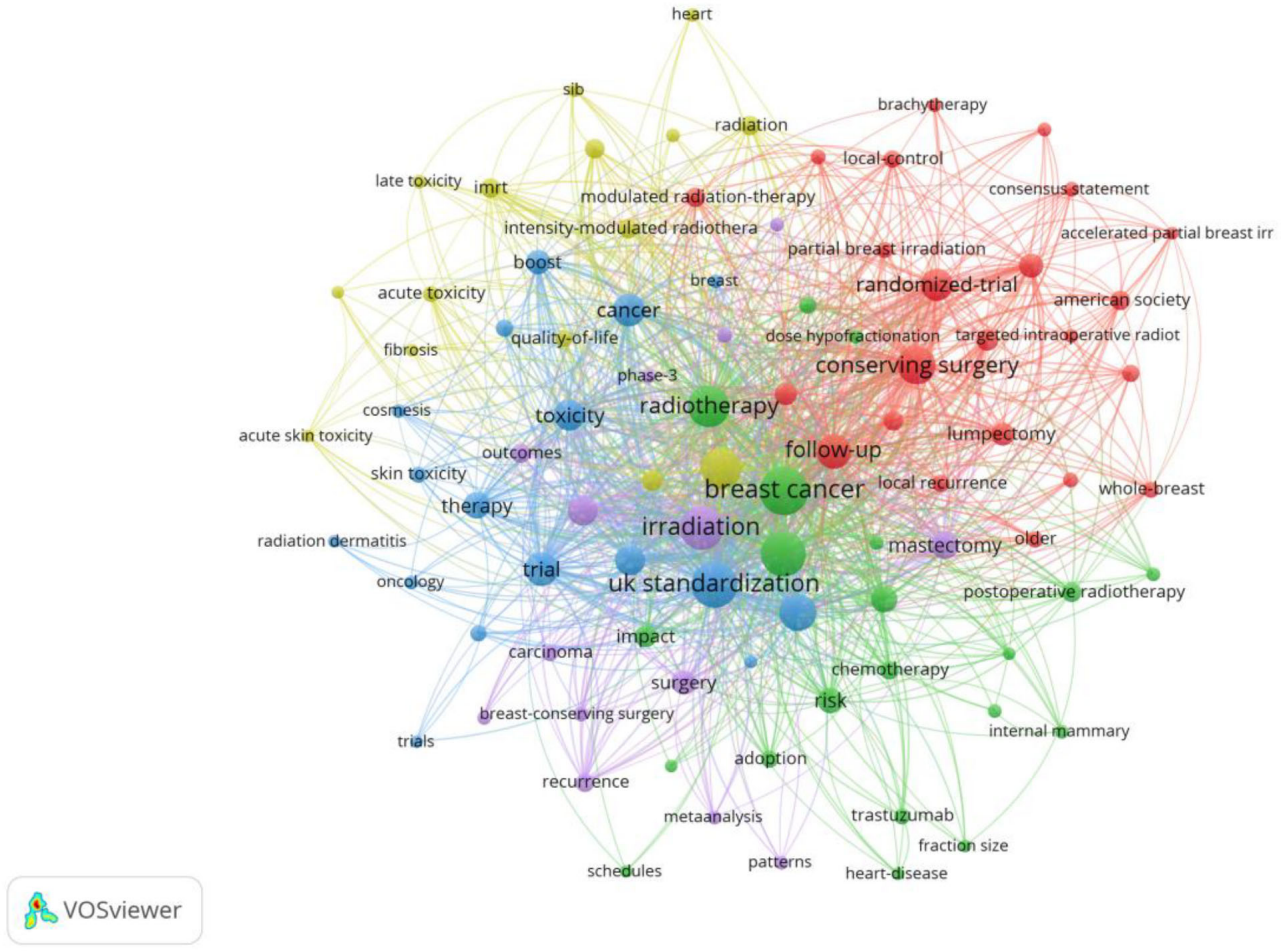
Keyword co-occurrence visualization using VOSviewer (1.6.20).

**Figure 7:**
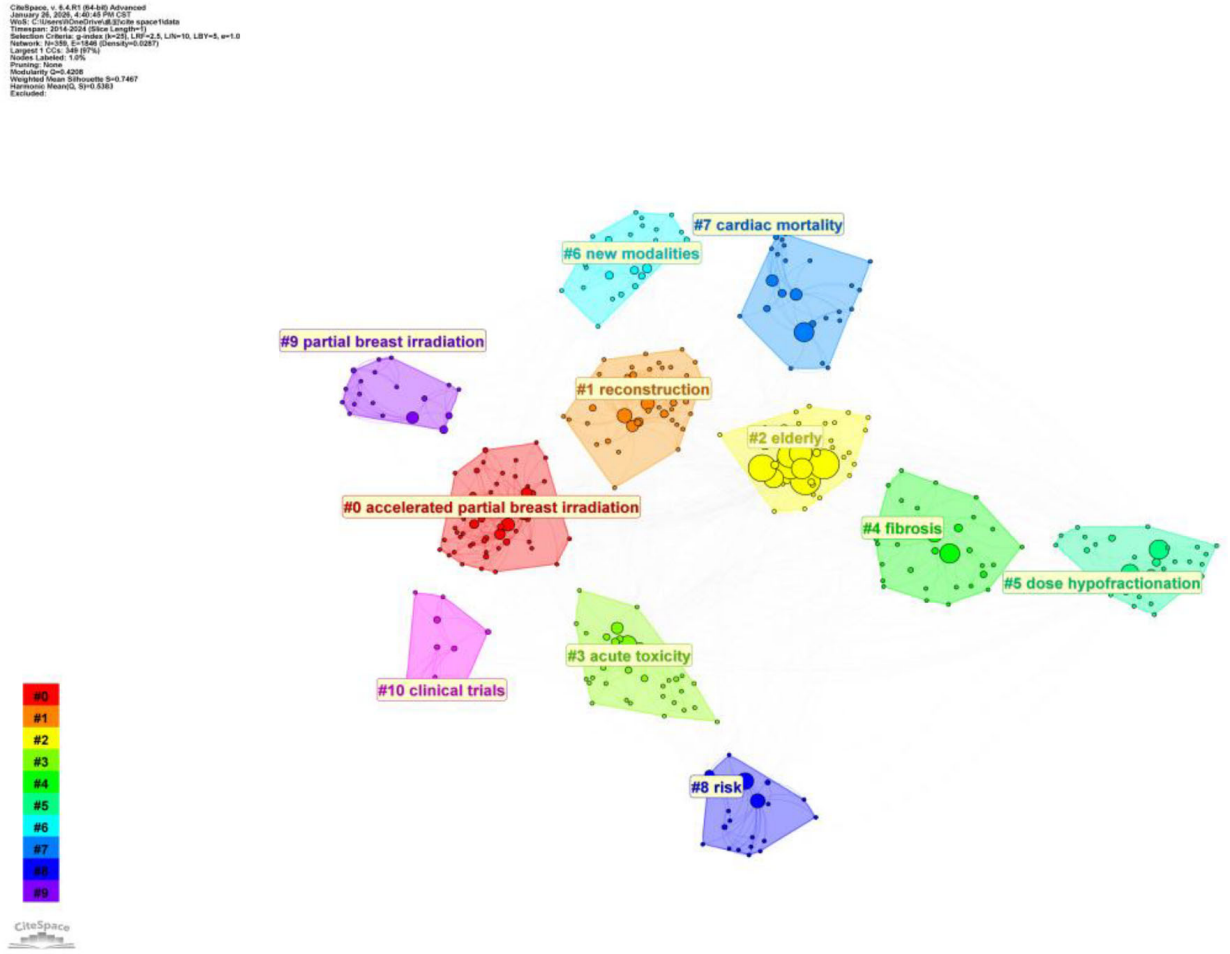
Keyword clustering using CiteSpace (6.4.R1).

**Figure 8:**
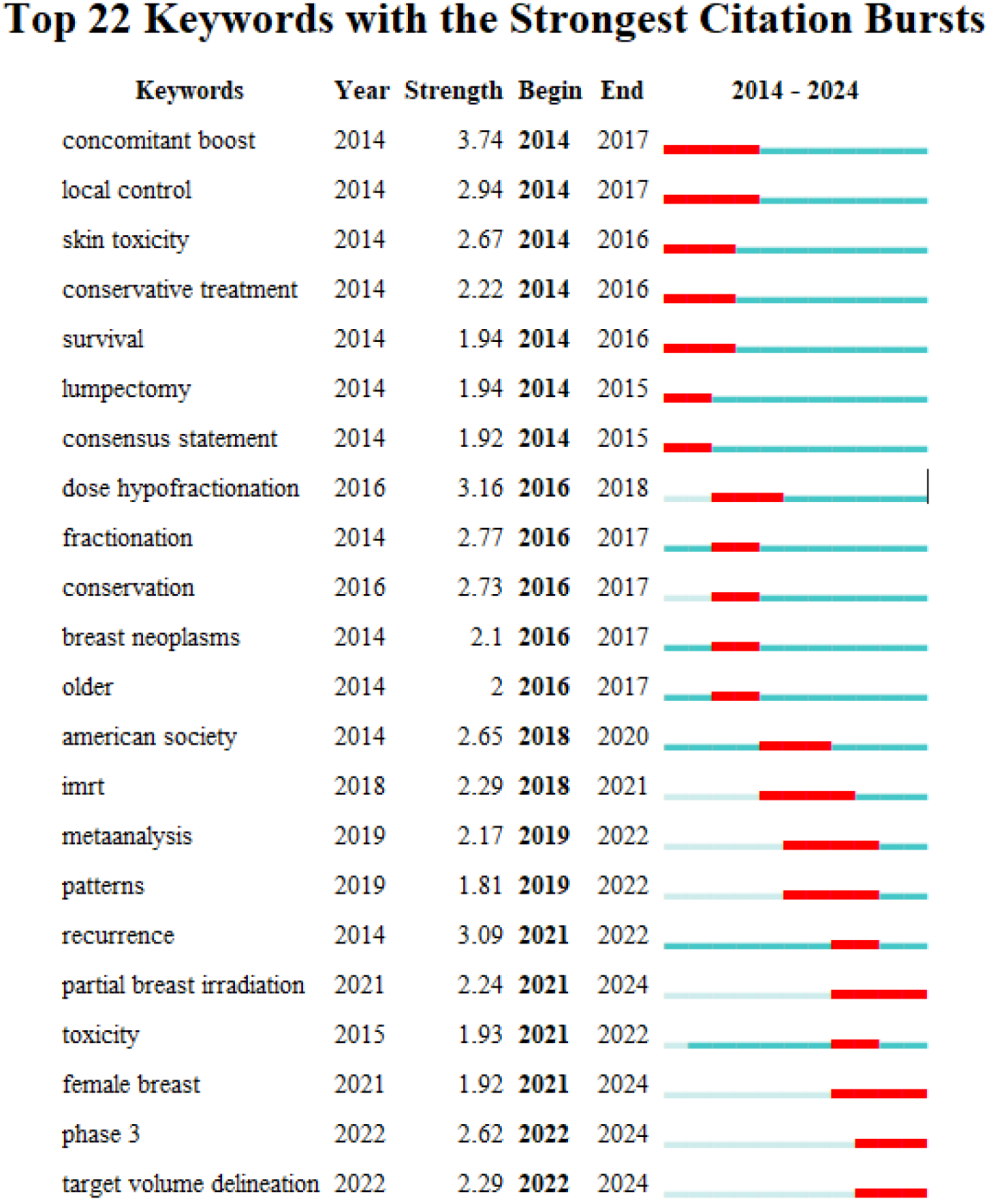
Keyword emergence/burst analysis using CiteSpace (6.4.R1).

Using CiteSpace (6.4.R1) and VOSviewer (1.6.20) software, keywords were visualized through co-occurrence analysis, clustering, and highlighting, yielding **Figures 6**–**8**.

**Figure 6** shows the keyword co-occurrence network generated using VOSviewer, illustrating the relationships among high-frequency keywords in research on hypofractionated radiotherapy for breast cancer. The network is characterized by several densely connected keyword clusters, indicating strong thematic associations across clinical efficacy, toxicity, treatment techniques, and patient-related outcomes. Central keywords such as “breast cancer,” “radiotherapy,” “irradiation,” and “hypofractionation” occupy prominent positions in the network, reflecting their foundational role in this research field. Closely connected terms—including “toxicity,” “local control,” “quality of life,” “partial breast irradiation,” and “conserving surgery”—suggest that current research emphasizes not only oncological outcomes but also treatment safety and patient-centered endpoints. The dense interconnections among keywords related to toxicity (e.g., skin toxicity, fibrosis, cardiac effects) and treatment techniques (e.g., IMRT, boost, target volume) highlight the integrated nature of technical optimization and clinical outcome evaluation in hypofractionated radiotherapy research.

To further clarify the thematic structure of the field, CiteSpace was used to perform keyword clustering analysis (**Figure 7**). The clustering results demonstrated good structural validity, with a modularity value (Q) of 0.4208 and a weighted mean silhouette score (S) of 0.7467, indicating reliable and well-defined clusters. A total of 11 major keyword clusters were identified. The largest cluster, #0 “accelerated partial breast irradiation,” represents a core research hotspot focused on treatment de-escalation, reduced target volumes, and shortened radiotherapy schedules. Cluster #1 “reconstruction” reflects increasing attention to hypofractionated radiotherapy in patients undergoing breast reconstruction, an area characterized by growing clinical interest but limited high-level evidence. Patient subgroup–specific research is represented by #2 “elderly,” emphasizing treatment tolerance and convenience, while safety-related themes are captured by #3 “acute toxicity” and #4 “fibrosis,” addressing short-term and late adverse effects, respectively. Cluster #5 “dose hypofractionation” highlights ongoing efforts to refine fractionation regimens, whereas #6 “new modalities” reflects technological innovation and emerging treatment approaches. Long-term safety concerns are particularly evident in #7 “cardiac mortality,” underscoring persistent uncertainties regarding cardiotoxicity. Risk stratification and outcome prediction are represented by #8 “risk,” while localized treatment strategies are further explored in #9 “partial breast irradiation.” Finally, #10 “clinical trials” emphasizes the central role of prospective studies in validating hypofractionated radiotherapy strategies.

**Figure 8** presents the top 22 keywords with the strongest citation bursts, illustrating the temporal evolution of research focus from 2014 to 2024. Early burst keywords (2014–2017), such as “local control,” “skin toxicity,” “conservative treatment,” “lumpectomy,” and “consensus statement,” reflect the initial emphasis on validating clinical efficacy and short-term safety. During the intermediate period (2018–2020), burst terms including “dose hypofractionation,” “IMRT,” and “American Society” indicate growing attention to dose optimization, advanced radiotherapy techniques, and guideline development. In more recent years (2021–2024), emerging burst keywords such as “partial breast irradiation,” “toxicity,” “female breast,” “phase 3,” and “target volume delineation” highlight a shift toward precision treatment, late toxicity assessment, and high-level evidence generation. Overall, the burst analysis demonstrates a clear transition from early efficacy validation toward refined treatment strategies, patient-specific optimization, and long-term safety evaluation, mirroring the maturation of hypofractionated radiotherapy as a guideline-supported standard of care in breast cancer (38–40).

### 3.5 Highly cited literature

**Figure 9:**
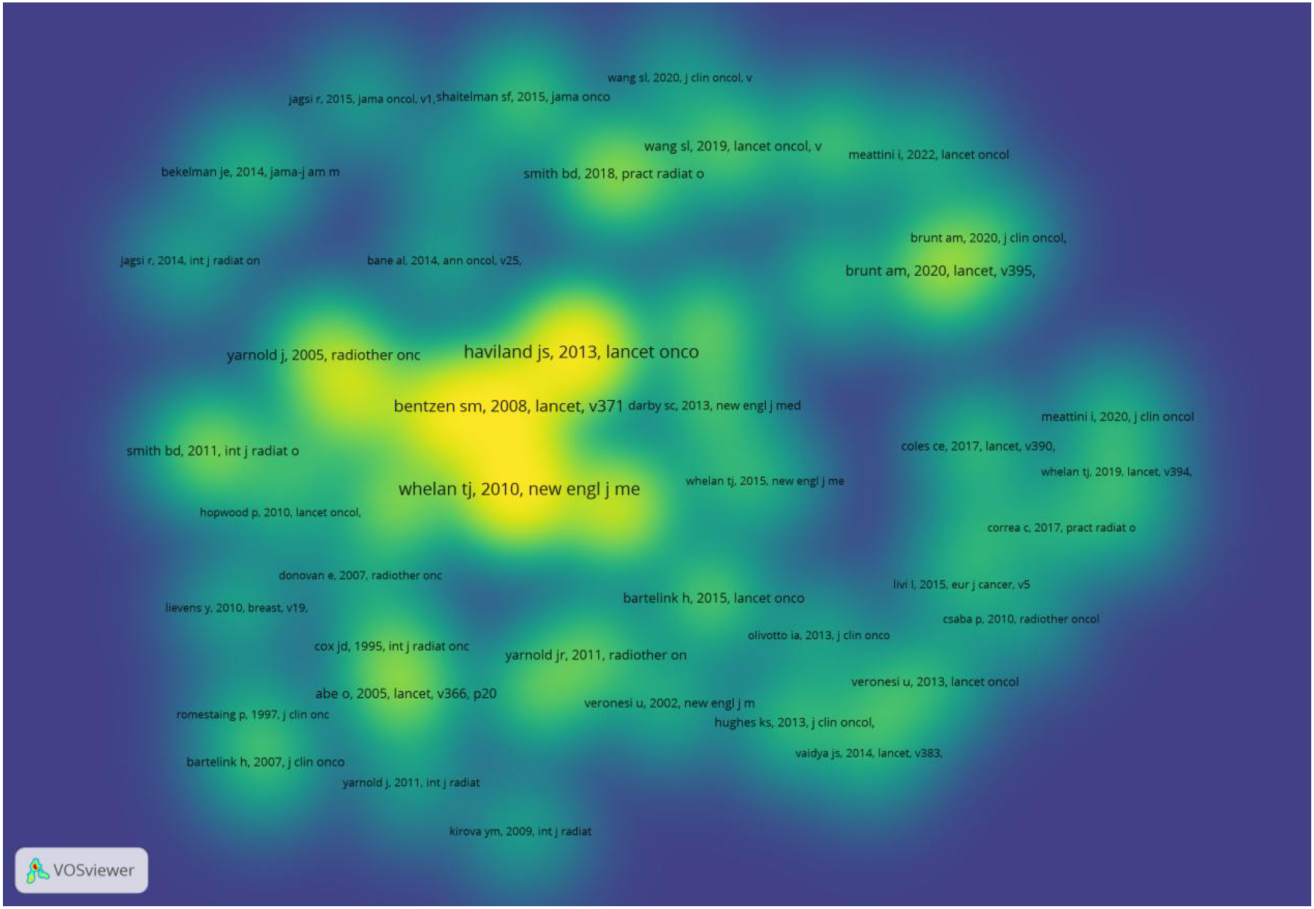
Analysis of highly cited literature using VOSviewer (1.6.20).

Visual analysis of highly cited references (≥ citations) using VOSviewer (1.6.20) yielded 224 documents, as shown in **Figure 9**.

The top three most cited publications were: Whelan TJ et al. (2010) in The New England Journal of Medicine (138 citations), Haviland JS et al. (2013) in The Lancet Oncology (125 citations), and Bentzen SM et al. (2008) in The Lancet (104 citations).

Ranked first is the 2010 study by Whelan TJ et al., published in the New England Journal of Medicine, a landmark achievement in the field of high-dose fractionated radiotherapy for early-stage breast cancer. This multicenter randomized controlled trial enrolled 1,234 patients with early-stage breast cancer (T1-2N0-1) following breast-conserving surgery. Participants were randomized to either the hypofractionated radiotherapy group (42.5 Gy in 16 fractions, completed in 3.2 weeks) or the conventional fractionation group (50Gy/25 fractions, completed in 5 weeks). The primary endpoint was the 5-year local recurrence rate. Results showed no significant statistical difference in 5-year local recurrence rates between groups (6.2% vs 5.9%, P = 0.83), with no apparent differences in distant metastasis rates, overall survival, or disease-free survival. This study provided the first large-scale validation that high-dose fractionated radiotherapy after breast-conserving surgery is non-inferior to conventional fractionation, offering key evidence to support clinical guideline recommendations. Ranked second is the 2013 study by Haviland JS et al. published in The Lancet Oncology, presenting long-term follow-up results from the UK START B trial. This study enrolled 2,236 patients with early-stage breast cancer (including those who underwent breast-conserving or radical surgery) to compare the efficacy of three radiotherapy regimens: conventional fractionation (50 Gy/25 fractions), intermediate fractionation (40 Gy/15 fractions), and hyperfractionated fractionation (36 Gy/12 fractions). After a median follow-up of 10 years, the median local recurrence rate in the intermediate fractionation group (5.2%) showed no significant difference compared to the conventional fractionation group (5.0%) (P = 0.79). Although the rate in the hyperfractionated group (6.8%) was slightly higher, it remained within an acceptable range. Furthermore, no significant statistical differences were observed in long-term toxicities (e.g., fibrosis, breast retraction) among the three groups. This study supports incorporating medium-fractionation regimens (e.g., 40 Gy/15 fractions) into standard treatment options, further solidifying the clinical position of high-fractionation radiotherapy. Ranked third is the 2008 study by Bentzen SM et al. published in The Lancet, which provided crucial theoretical support for high-fractionation radiotherapy from a radiobiological perspective. Based on the Linear-Quadratic (LQ) model, the analysis indicated that breast cancer cells exhibit a relatively low α/β ratio (approximately 3 Gy), making them less sensitive to changes in fractionated doses. Consequently, appropriately reducing the total dose during high-fractionation radiotherapy does not compromise efficacy. This theory clearly elucidates the biological mechanism underpinning the suitability of high-fractionation radiotherapy for breast cancer, providing crucial evidence for designing clinical dose regimens (38, 39, 41–43).

### 3.6 Clinical progress analysis

To assess the evolution of clinical evidence supporting hypofractionated radiotherapy, clinical trials were identified through a structured search of the PubMed database. The search strategy combined terms related to breast cancer and hypofractionated radiotherapy. Eligible studies were screened based on predefined criteria, including prospective study design, relevance to breast cancer radiotherapy, and availability of clinical outcome data. Randomized controlled trials and large prospective studies were prioritized. Each included trial was reviewed to extract information on study design, patient population, fractionation regimen, follow-up duration, and key oncological and toxicity outcomes. This process allowed a qualitative assessment of the strength and maturity of clinical evidence in parallel with bibliometric findings.

We identified 6 clinical trials from the PubMed database to evaluate the development of the field of hypofractionated radiotherapy for breast cancer, mainly with the following 6 arguments (44–47):

1. Compared with conventional fractionation radiotherapy, large-fraction radiotherapy demonstrated non-inferiority in 5-year local-regional recurrence rates (absolute difference 0.2%, 90% CI -3.0 to 2.6; non-inferiority p < 0.0001) and a lower incidence of grade 3 acute skin toxicity (3% vs 8%, p < 0.0001).
2. Both 5 fractions of 27 Gy and 26 Gy achieved non-inferiority for 5-year ipsilateral breast tumor recurrence compared to 15 fractions of 40 Gy (27 Gy absolute difference -0.3%, p=0.0022; 26 Gy absolute difference -0.7%, p=0.00019).
3. Compared with 15 fractions of 40 Gy, 5 fractions of 27 Gy radiotherapy carried a higher risk of moderate/severe normal tissue effects in the breast/chest wall (hazard ratio 1.55, 95% CI 1.32 to 1.83, p < 0.0001), whereas 26 Gy radiotherapy showed no significant difference compared with 40 Gy (hazard ratio 1.12, p = 0.20).
4. Compared with delayed short-course radiotherapy and delayed long-course radiotherapy, short-course radiotherapy showed no significant difference in the risk of local recurrence (hazard ratios 1.44 and 2.24, respectively; p=0.48), meeting non-inferiority criteria.
5. Delayed short-course radiotherapy significantly reduced postoperative complication risk compared with short-course radiotherapy (HR 0.61, 95% CI 0.45 to 0.83, p=0.001).
6. Breast cancer patients under 45 years old receiving the HF regimen demonstrated higher psychosocial-behavioral scores at 6 months (23.6 vs 22.0, p=0.047), experienced lower levels of adverse reactions (e.g., nausea), and had lower rates of treatment interruption (2.7% vs 7.7%, p=0.03) and need for unpaid leave (8.5% vs 16.9%, p=0.02).

## 4 Discussion

This study provides the first bibliometric knowledge-mapping analysis of hypofractionated radiotherapy (HFRT) for breast cancer that explicitly addresses the mismatch between research activity and clinical evidence adoption. By analyzing global literature from 2014 to 2024, we identified a three-phase development trajectory, key international collaborations, and evolving research hotspots. Importantly, we revealed persistent “research–evidence gaps”—areas where scientific output has grown rapidly but guideline-level or practice-changing evidence remains insufficient.

Our findings highlight three critical gaps: (i) inadequate long-term data on cardiac and pulmonary safety, (ii) limited assessment of cosmetic and patient-reported outcomes in younger populations, and (iii) insufficient high-quality evidence for HFRT use in complex clinical scenarios, such as locally advanced disease or post-reconstruction patients. These gaps explain why, despite broad acceptance of HFRT for early-stage breast cancer, clinical guidelines still adopt a cautious or conditional stance in certain subgroups.

Summary of Research–Evidence Gaps in Hypofractionated Radiotherapy for Breast Cancer.

**Figure 10:**
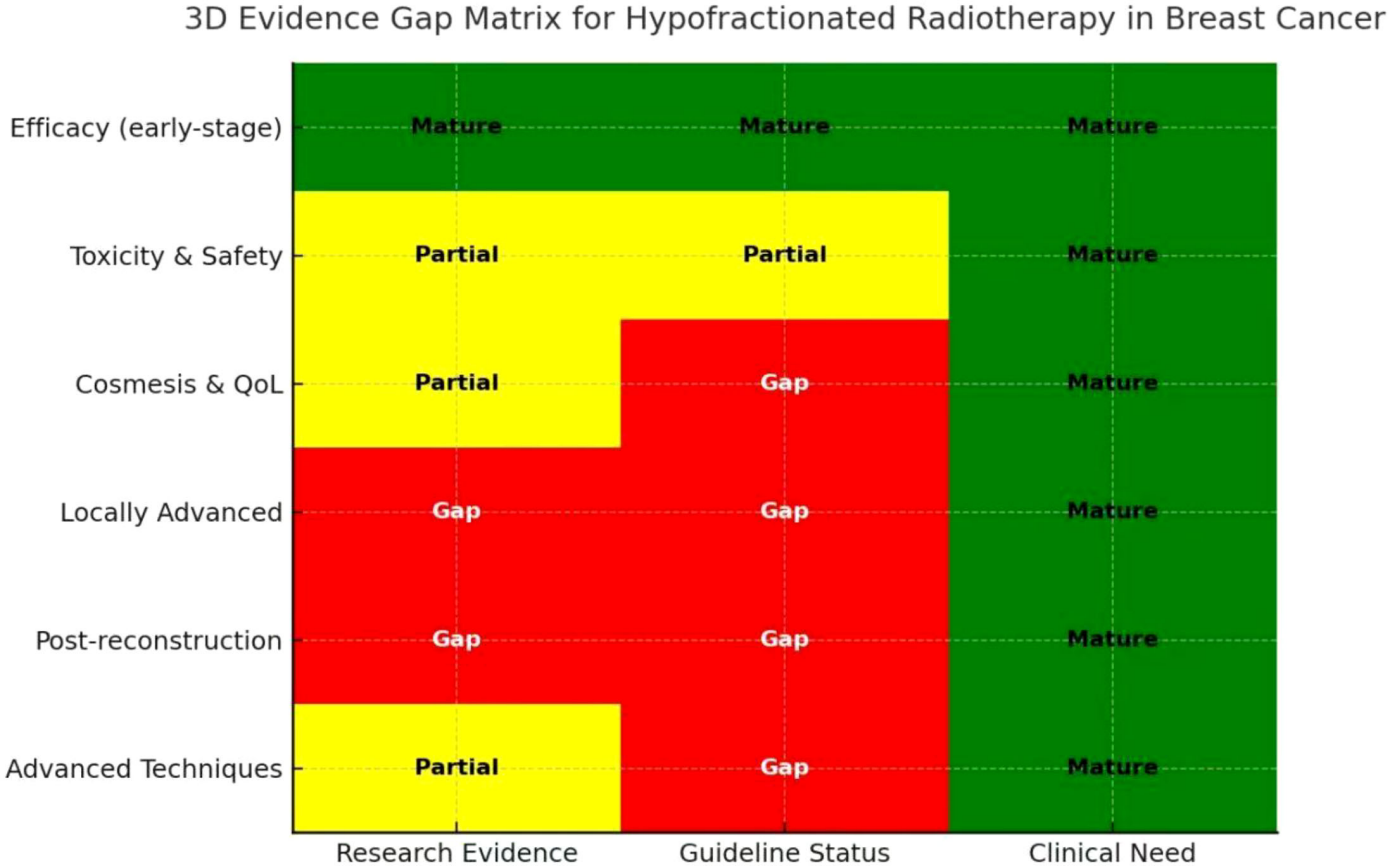
Evidence gap matrix of hypofractionated radiotherapy for breast cancer: alignment of research evidence, guideline position, and clinical needs.

To understand the evidence gaps in hypofractionated radiotherapy for breast cancer, we can refer to the 3D Evidence Gap Matrix. For details, please see **Figure 10**, which aligns research evidence, guideline status, and clinical need.

The Evidence Gap Matrix presented in **Figure 10** is intended as a conceptual mapping framework that integrates bibliometric trends, clinical evidence availability, and guideline positions. It does not represent a formal evidence-ranking system and should not be interpreted as an objective measure of evidence strength.

Research Hotspot→Current Evidence Status→Guideline Position (ASTRO/ESTRO/NCCN/CSCO)→Identified Evidence Gap.

Efficacy of HFRT in early-stage breast cancer. Multiple RCTs (START, FAST-Forward, Whelan) confirm non-inferiority to CFRT. Strong recommendation for early-stage, breast-conserving therapy. Evidence is mature, with no major gaps.

Toxicity and safety: Acute toxicity is well documented; long-term follow-up is limited. Guidelines note the need for monitoring late cardiac/pulmonary effects. Lack of >10-year prospective data on cardiac and pulmonary toxicity (48).

Cosmetic outcomes & quality of life. Some studies (e.g., Lozza, RAPID trial) show benefit, but are inconsistent across age groups. Guidelines rarely specify cosmetic/QoL as decision factors. Limited standardized assessment in younger patients and long-term cosmetic outcomes (49).

Locally advanced breast cancer: Few high-quality trials; mainly retrospective or single-institution data. Guidelines cautious, CFRT still standard. Insufficient RCT evidence to support HFRT in locally advanced disease (50).

Post-reconstruction patients: Sparse literature; case series only. Guidelines do not recommend HFRT routinely. No robust evidence on safety/cosmesis after reconstruction (51).

Integration with advanced techniques (VMAT, DIBH, SGRT, AI, radiomics). Planning/dosimetric studies suggest reduced organ-at-risk dose. Not yet incorporated into guidelines. Need multicenter clinical validation and long-term outcome data (52).

By integrating bibliometric trends with guideline evolution, this study goes beyond traditional citation analysis and offers a strategic roadmap for aligning research priorities with clinical needs. Such alignment is essential to accelerate evidence translation, optimize treatment personalization, and ensure that future guideline updates are grounded in robust, practice-relevant data.

Beyond the specific clinical context of breast cancer, the bibliometric and knowledge-mapping framework applied in this study has broader methodological implications for oncology and radiation therapy research. By integrating publication trends, keyword co-occurrence, clustering analysis, citation bursts, and clinical evidence mapping, this approach enables a structured assessment of how research activity aligns with evidence generation and guideline adoption. This framework can be readily extended to other tumor sites and radiotherapy modalities, such as prostate cancer, lung cancer, head and neck cancer, or emerging techniques including stereotactic body radiotherapy and proton therapy. In these settings, the same methodology may be used to identify research hotspots, trace evidence maturation, and highlight areas where clinical practice has advanced faster—or slower—than high-level evidence. Therefore, this approach provides a transferable tool for evaluating research–evidence gaps and informing future trial design and guideline development across diverse oncologic disciplines.

This framework can be readily extended to other tumor sites and radiotherapy modalities, such as prostate cancer, lung cancer, head and neck cancer, or emerging techniques including stereotactic body radiotherapy and proton therapy. In these settings, the same methodology may be used to identify research hotspots, trace evidence maturation, and highlight areas where clinical practice has advanced faster—or slower—than high-level evidence. Therefore, this approach provides a transferable tool for evaluating research–evidence gaps and informing future trial design and guideline development across diverse oncologic disciplines.

To address the identified research–evidence gaps, future studies should prioritize multicenter prospective designs to enhance statistical power, improve generalizability, and enable robust subgroup analyses in underrepresented clinical settings. The integration of artificial intelligence and radiomics into trial design represents a feasible pathway to refine patient selection and personalize fractionation strategies, provided these tools are evaluated alongside clinically meaningful endpoints. However, the translation of emerging evidence into practice remains challenged by several barriers, including limited funding for long-term follow-up studies, constraints on data sharing across institutions and countries, and delays inherent to clinical guideline update cycles. Coordinated efforts among investigators, funding agencies, and guideline committees will therefore be essential to accelerate evidence generation and clinical adoption (53–55).

### 4.1 Limitations

This study has several limitations that should be acknowledged.

Firstly, although the included clinical trials provide valuable evidence supporting the efficacy and safety of hypofractionated radiotherapy, most landmark randomized controlled trials predominantly enrolled patients with early-stage breast cancer. As a result, the generalizability of these findings to patients with locally advanced disease, postmastectomy settings, or post-reconstruction radiotherapy remains limited.

Secondly, while oncological outcomes such as local control and survival are well documented, long-term follow-up data on cardiac and pulmonary toxicity—particularly beyond 10 years—are relatively scarce. Given the potential for late radiation-related adverse effects, this limitation restricts a comprehensive assessment of long-term safety.

Thirdly, heterogeneity exists among trials with respect to fractionation regimens, boost strategies, target volume definitions, and toxicity assessment methods, which complicates direct comparison across studies. In addition, patient-reported outcomes and cosmetic assessments are inconsistently reported and often underrepresented, especially in younger patient populations.

Finally, the clinical trial evidence summarized in this study was reviewed qualitatively rather than through a formal systematic review or evidence-grading framework. Therefore, the strength of evidence should be interpreted in conjunction with existing guidelines and consensus statements.

## 5 Conclusion

Hypofractionated radiotherapy has evolved from an emerging alternative to a well-established, evidence-supported standard for early-stage breast cancer. Over the past decade, global research has expanded rapidly, and multiple high-quality randomized trials have confirmed its non-inferiority in terms of tumor control and acute toxicity. However, critical gaps remain in long-term safety, cosmetic and quality-of-life outcomes, and applications to complex patient groups such as locally advanced disease or post-reconstruction cases.

To fully realize the benefits of HFRT, future research should prioritize prospective multicenter studies with extended follow-up, standardized patient-reported outcome measures, and integration of radiomics and AI-based analytic frameworks (56–59). Such efforts will help refine patient selection, enhance treatment precision, and accelerate alignment between research activity, clinical evidence, and guideline recommendations (60, 61). Ultimately, closing these evidence gaps will advance HFRT toward a fully personalized and patient-centered standard of care.

## Data Availability

The original contributions presented in the study are included in the article/supplementary material. Further inquiries can be directed to the corresponding authors.
